# Metformin requires 4E-BPs to induce apoptosis and repress translation of Mcl-1 in hepatocellular carcinoma cells

**DOI:** 10.18632/oncotarget.10671

**Published:** 2016-07-18

**Authors:** Mamatha Bhat, Akiko Yanagiya, Tyson Graber, Nataliya Razumilava, Steve Bronk, Domenick Zammit, Yunhao Zhao, Chadi Zakaria, Peter Metrakos, Michael Pollak, Nahum Sonenberg, Gregory Gores, Maritza Jaramillo, Masahiro Morita, Tommy Alain

**Affiliations:** ^1^ Goodman Cancer Centre, Department of Biochemistry, McGill University, Montreal, Canada; ^2^ Division of Gastroenterology, University Health Network and University of Toronto, Toronto, Canada, USA; ^3^ Division of Gastroenterology and Hepatology, Mayo Clinic, Rochester, Minnesota; ^4^ Children's Hospital of Eastern Ontario Research Institute, Department of Biochemistry, Microbiology and Immunology, University of Ottawa, Ottawa, Ontario, Canada; ^5^ Division of Gastroenterology, University of Michigan, Ann Arbor, Michigan, USA; ^6^ Departments of Medicine and Oncology, Lady Davis Institute for Medical Research and Segal Cancer Center, Montreal, Canada; ^7^ Department of Surgery, McGill University Health Centre, Montreal, Canada; ^8^ INRS Institut Armand-Frappier Research Centre, Laval, Quebec, Canada

**Keywords:** metformin, hepatocellular carcinoma, mRNA translation, mTORC1, 4E-BPs

## Abstract

Metformin inhibits the mammalian target of rapamycin complex 1 (mTORC1) signaling pathway, which is frequently upregulated in hepatocellular carcinoma (HCC). Metformin has also been shown to induce apoptosis in this cancer. Here, we investigate whether metformin-induced apoptosis in HCC is mediated by the downstream mTORC1 effectors eukaryotic initiation factor 4E and (eIF4E)-binding proteins (4E-BPs). Further, we ask whether changes in 4E-BPs activity during metformin treatment negatively regulate translation of the anti-apoptotic *myeloid cell leukemia 1* (*Mcl-1*) mRNA. A genetic HCC mouse model was employed to assess the ability of metformin to reduce tumor formation, induce apoptosis, and control 4E-BP1 activation and Mcl-1 protein expression. In parallel, the HCC cell line Huh7 was transduced with scrambled shRNA (control) or shRNAs targeting 4E-BP1 and 4E-BP2 (4E-BP knock-down (KD)) to measure differences in mRNA translation, apoptosis, and Mcl-1 protein expression after metformin treatment. In addition, immunohistochemical staining of eIF4E and 4E-BP1 protein levels was addressed in a HCC patient tissue microarray. We found that metformin decreased HCC tumor burden, and tumor tissues showed elevated apoptosis with reduced Mcl-1 and phosphorylated 4E-BP1 protein levels. In control but not 4E-BP KD Huh7 cells, metformin induced apoptosis and repressed *Mcl-1* mRNA translation and protein levels. Immunostaining of HCC patient tumor tissues revealed a varying ratio of eIF4E/4E-BP1 expression. Our results propose that metformin induces apoptosis in mouse and cellular models of HCC through activation of 4E-BPs, thus tumors with elevated expression of 4E-BPs may display improved clinical chemopreventive benefit of metformin.

## INTRODUCTION

Hepatocellular carcinoma (HCC) is the most rapidly rising cause of cancer-related deaths among men in the United States [1]. The incidence of HCC has been rising across North America, with increasing prevalence of cirrhosis secondary to hepatitis C or non-alcoholic steatohepatitis [2–4]. HCC is frequently diagnosed at an advanced stage, when curative treatments such as resection or liver transplant are no longer feasible, resulting in a poor overall 5-year survival rate of less than 15% [5]. Mutations in genes encoding for upstream regulators of mTORC1, such as PI3K, PTEN, AKT and TSC, are frequently observed in HCC, resulting in a hyperactivated mTORC1 signaling pathway in up to 50% of HCC tumors [6].

The mTORC1 pathway is stimulated by growth factors such as the insulin growth factor (IGF-1), and affected by cellular energetics [7, 8]. AMP-activated protein kinase (AMPK), an intracellular sensor serving to maintain energy balance, is stimulated by increased energy consumption as reflected by an elevated AMP/ATP ratio. AMPK activation in turn inhibits mTORC1 activity and anabolic processes, including the energy-consuming process of protein synthesis [9, 10]. Metformin, a biguanide drug commonly used to treat type 2 diabetes, has been shown to inhibit the mTORC1 pathway through two mechanisms: it induces AMPK activation, and decreases Insulin Growth Factor (IGF-1)-mediated stimulation of the PI3K/Akt/mTOR pathway [11–13]. Interestingly, metformin has previously been found to inhibit tumor growth *in vitro* and *in vivo* by inducing apoptosis in various malignancies including breast [14–16], lung [17, 18] and melanoma [19, 20]. Furthermore, retrospective studies have suggested that metformin prevents development of HCC among diabetic patients with chronic liver disease [21–30]. Therefore, chemoprevention with metformin could be most effective where insulin resistance is present and the mTORC1 pathway is upregulated, such as in diabetic and obese patients with chronic liver disease.

mTORC1 primarily regulates cap-dependent mRNA translation through the phosphorylation of 4E-BPs (of which 3 members exist 4E-BP1, 4E-BP2, and 4E-BP3). Phosphorylated 4E-BPs no longer bind eIF4E, which allows the association of eIF4E with eIF4G to form the eIF4F complex and initiate translation [31, 32]. When mTORC1 is inactive, hypophosphorylated 4E-BPs act as translation repressors by binding eIF4E and preventing its association with eIF4G. Levels of 4E-BPs, and importantly phosphorylated 4E-BPs, vary in tumors, while eIF4E is frequently found overexpressed in a number of malignancies including HCC [33]. Increased levels of eIF4E in malignancies are known to selectively enhance the translation of oncogenic mRNAs and fuel tumorigenesis [34], thereby making it especially pertinent to targeted therapy of HCC.

The aim of this study was to elucidate a mechanistic basis for the apoptotic effect of metformin on HCC. We found 1) that metformin induces apoptosis of HCC *in vitro* and in an established genetic mouse model of HCC, 2) that metformin treatment decreases expression of Mcl-1, an anti-apoptotic factor previously shown to be translationally regulated by mTORC1 activity [35], and 3) that these effects require the translation repressors 4E-BPs. Accordingly, liver cancer cells with reduced 4E-BP1/2 expression are more resistant to metformin-induced apoptosis, alongside sustained translation of *Mcl-1* mRNA and protein expression. Our results propose that apoptosis induced by metformin in liver cancer cells can be translationally controlled. As our tissue microarray shows that human HCCs can have varying levels of 4E-BP1 and eIF4E expression, this novel finding suggests a critical determinant by which metformin could prevent establishment of tumors in patients with chronic liver disease. Therefore, assessing the eIF4E/4E-BP1 ratio in tumors may help stratifying HCC patients that could benefit most from using metformin as a chemopreventive agent.

## RESULTS

### Metformin induces apoptosis of tumors in a HCC mouse model

**Figure 1:**
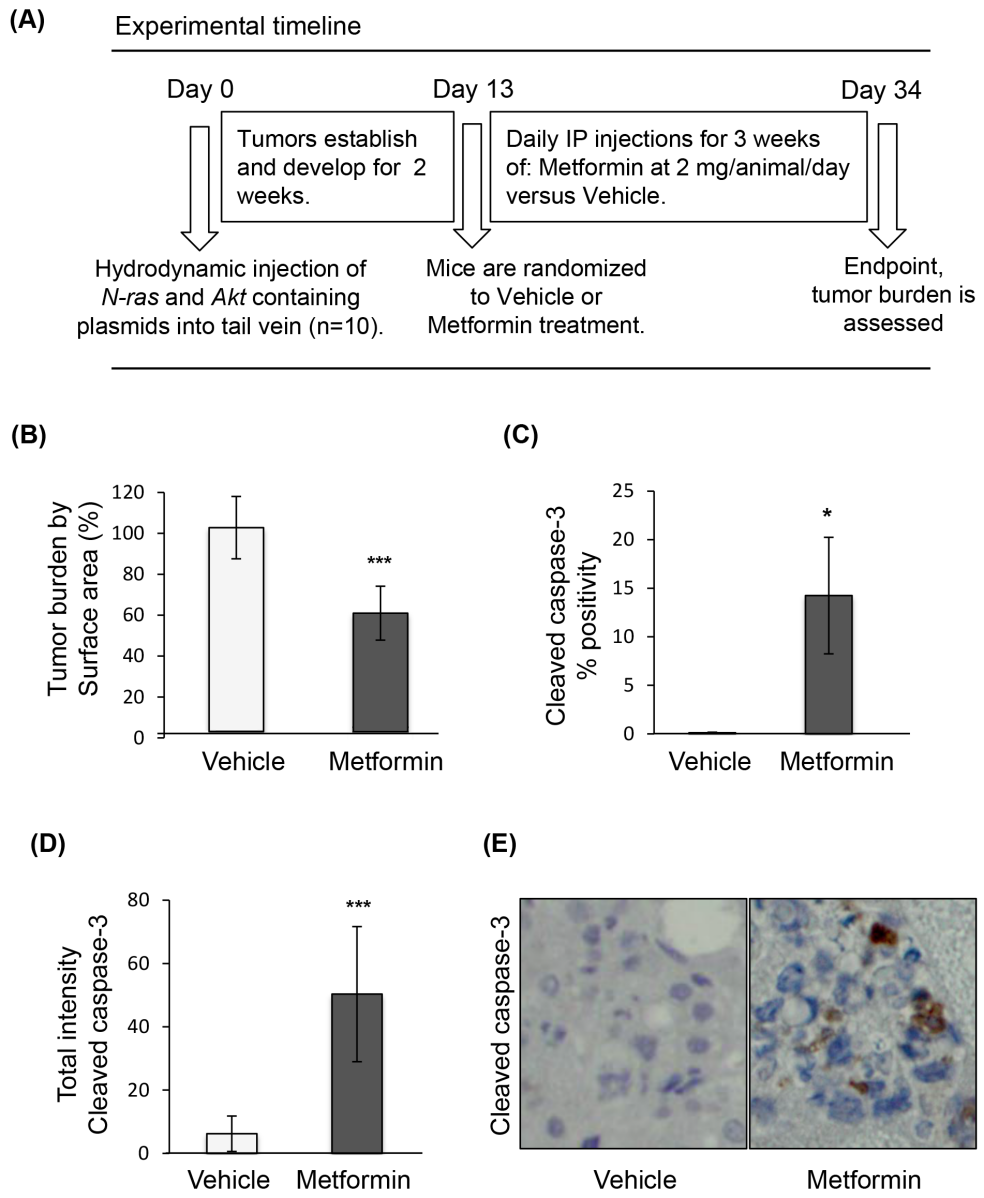
Metformin induces apoptosis of tumors in a genetic HCC mouse model **A**. Experimental timeline of treatment of the HCC mouse model; **B**. Surface area of tumor nodules from mice treated with metformin, expressed as percentage of surface area of tumor nodules from mice injected with vehicle (p=0.0002); **C**. Positivity (p=0.011) and **D**. intensity of cleaved caspase-3 expression (p=0.0003) in HCC tumors of mice treated with metformin, indicating induction of apoptosis by metformin; **E**. Representative images of HCC nodules of mice treated with vehicle or metformin (20X magnification), and stained for cleaved caspase-3.

**Figure 2:**
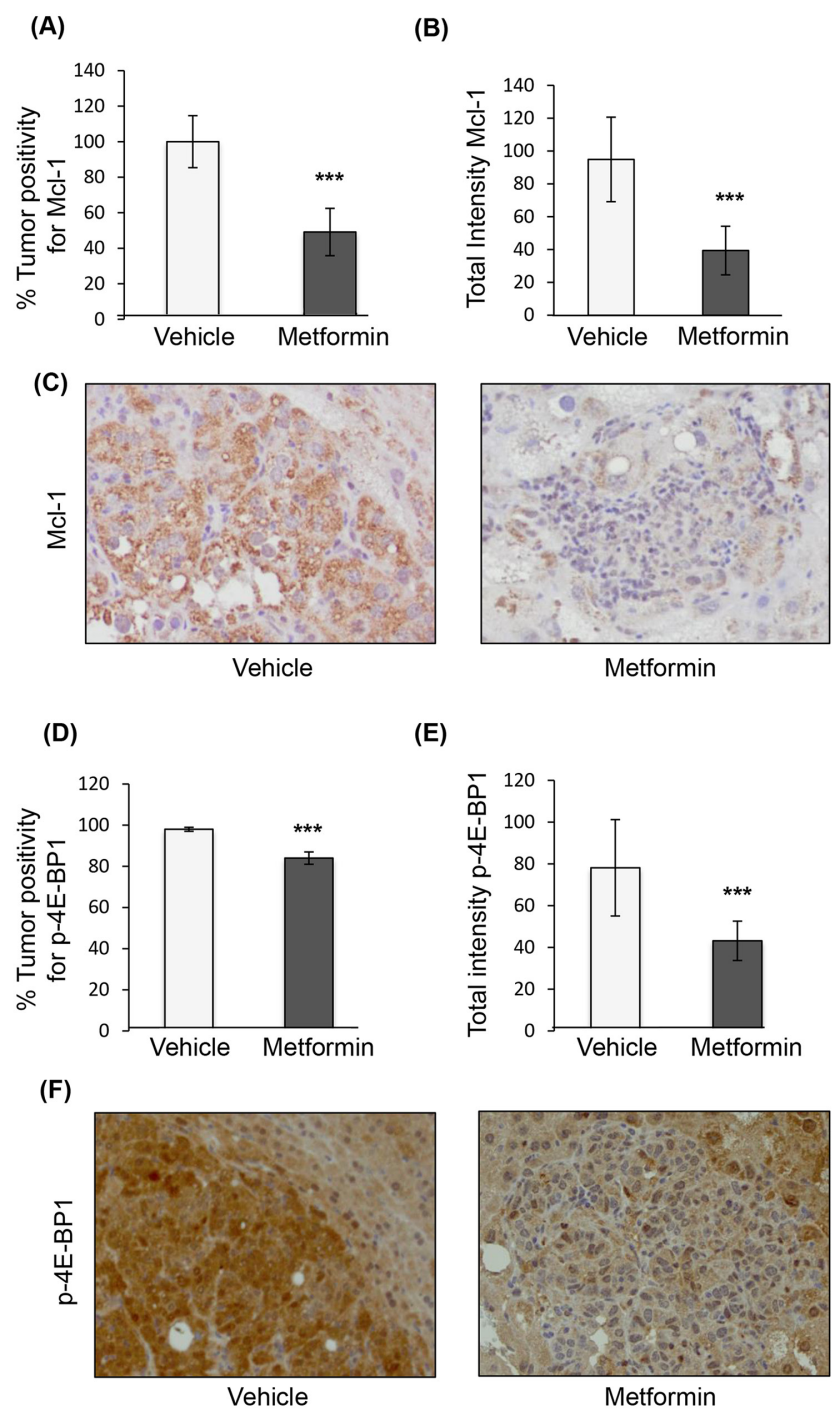
Immunohistochemical staining of nodules from the genetic mouse model of HCC after treatment with vehicle or metformin **A**. Positivity of Mcl-1 staining in tumors treated with metformin expressed as percentage of positivity normalized to nodules treated with vehicle (p<0.0001); **B**. Total intensity of Mcl-1 staining in tumors from mice treated with metformin as compared to those treated with vehicle (p=0.0003); **C**. Representative images of HCC nodules treated with vehicle or metformin (20X magnification) stained for Mcl-1; **D**. Positivity of phosphorylated 4E-BP1 (p-4E-BP1, Thr37/46) staining in tumors treated with metformin expressed as percentage of positivity of nodules from mice treated with vehicle (p=0.0002), and **E**. Total intensity positive of p-4E-BP1 staining (p<0.0001); **F**. Representative images of HCC nodules from mice treated with vehicle or metformin (20X magnification) stained for p-4E-BP1.

To examine the effect of metformin on HCC, we used an established HCC genetic mouse model (Figure 1A), in which hepatocarcinogenesis relies on upregulation of two key pathways, PI3K/Akt/mTOR and Ras/Raf/MAPK [36]. In this model, metformin treatment was associated with decreased tumor burden (p=0.0002) (Figure 1B), and presence of apoptosis in tumors, as evidenced by increased cleaved caspase-3 positive cells (Figure 1C) and total staining intensity (Figure 1D) of cleaved caspase-3 (p=0.011 and p=0.0003, respectively; Figure 1E shows a representative image of caspase-3 staining). Staining for the anti-apoptotic protein Mcl-1, whose expression was shown to be regulated by mTORC1 [35], revealed that there was a significant decrease in Mcl-1 positive cells in nodules of metformin-treated mice as compared to those from mice injected with vehicle alone (p<0.0001), as well as decreased intensity of Mcl-1 positive staining in metformin-treated nodules (p=0.0003) (Figures 2A-2C). The decrease in expression of Mcl-1 in the nodules correlated with reduced phosphorylation of 4E-BP1 (Thr37/46) in metformin-treated mice (Figure 2D–2F). Percentage positivity of phosphorylated 4E-BP1 staining was significantly diminished in the presence of metformin (p=0.0002), as was the total intensity of positive phosphorylated 4E-BP1 staining (p<0.0001). These results show that metformin treatment in a HCC mouse model limits HCC progression, increases tumor apoptosis, and reduces Mcl-1 and phosphorylated 4E-BP1 protein expression.

### Metformin decreases cell viability of HCC cell lines

**Figure 3:**
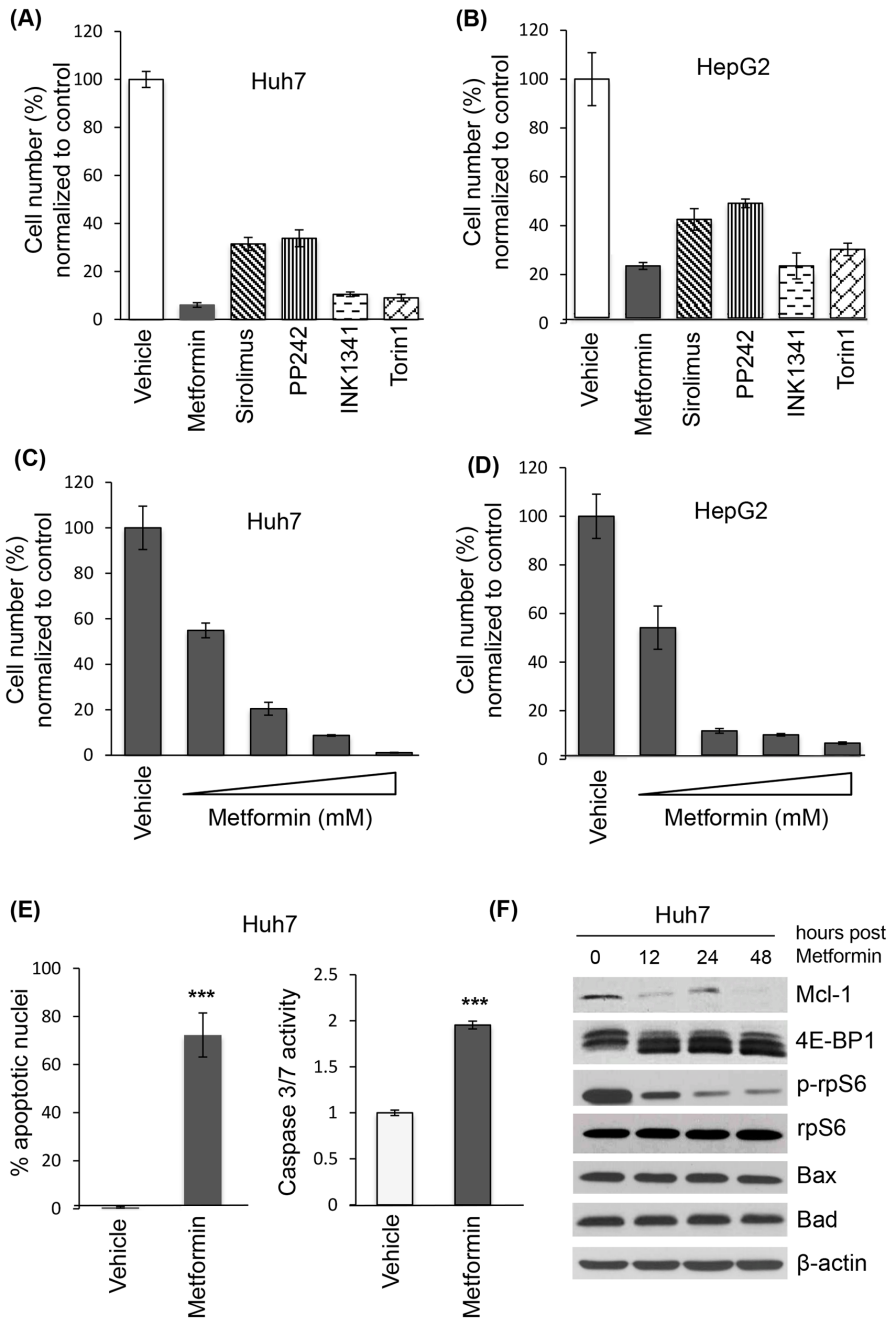
Metformin decreases cell viability, induces apoptosis of liver cancer cell lines and reduces Mcl-1 protein levels Trypan blue exclusion of Huh7 **(A)** and HepG2 **(B)** cells treated with metformin (10 mM), sirolimus (20 nM), and active site mTOR inhibitors PP242 (1 μM), INK1341 (250 nM) and Torin1 (250 nM) for 72 hours, expressed as percentage of vehicle-treated cells; Trypan blue exclusion of Huh7 **(C)** and HepG2 **(D)** cells treated with different metformin concentrations (0, 1, 5, 10, 20 mM) for 72 hours, expressed as percentage of vehicle-treated cells; **E**. Apoptotic measurements by DAPI and caspase fluorimetric assays of Huh7 cells treated with 10 mM metformin for 48 hours (p=0.0015 and p<0.0001 respectively); **F**. Western blot of indicated proteins from Huh7 lysates treated with 10 mM metformin for 0, 12, 24 or 48 hours. β-actin was used as a loading control.

To address the mechanism of metformin-induced cell death in HCC, we treated the HCC cell lines Huh7 and HepG2 with metformin and examined cell viability by trypan blue exclusion. Metformin decreased cell numbers at 72 hours in both cell lines, similar to active-site mTOR inhibitors (asTORi) INK1341 and Torin1 (Figures 3A and 3B). There was decreased viable cell number across metformin concentrations, with an IC_50_ of 1 mM (Figures 3C and 3D). DAPI staining and a fluorometric caspase 3/7 activity assay [37] confirmed that metformin significantly induced apoptosis at 48 hours (Figure 3E). A time course of metformin treatment also showed that expression of Mcl-1 protein decreased over time, correlated with decreased phosphorylation of the main downstream effectors of mTORC1, 4E-BP1 and ribosomal protein S6 (rpS6) (Figure 3F). In contrast, the expression of pro-apoptotic proteins Bad and Bax did not change over time.

### Metformin induces translational repression of the anti-apoptotic *Mcl-1* mRNA via 4E-BPs

**Figure 4:**
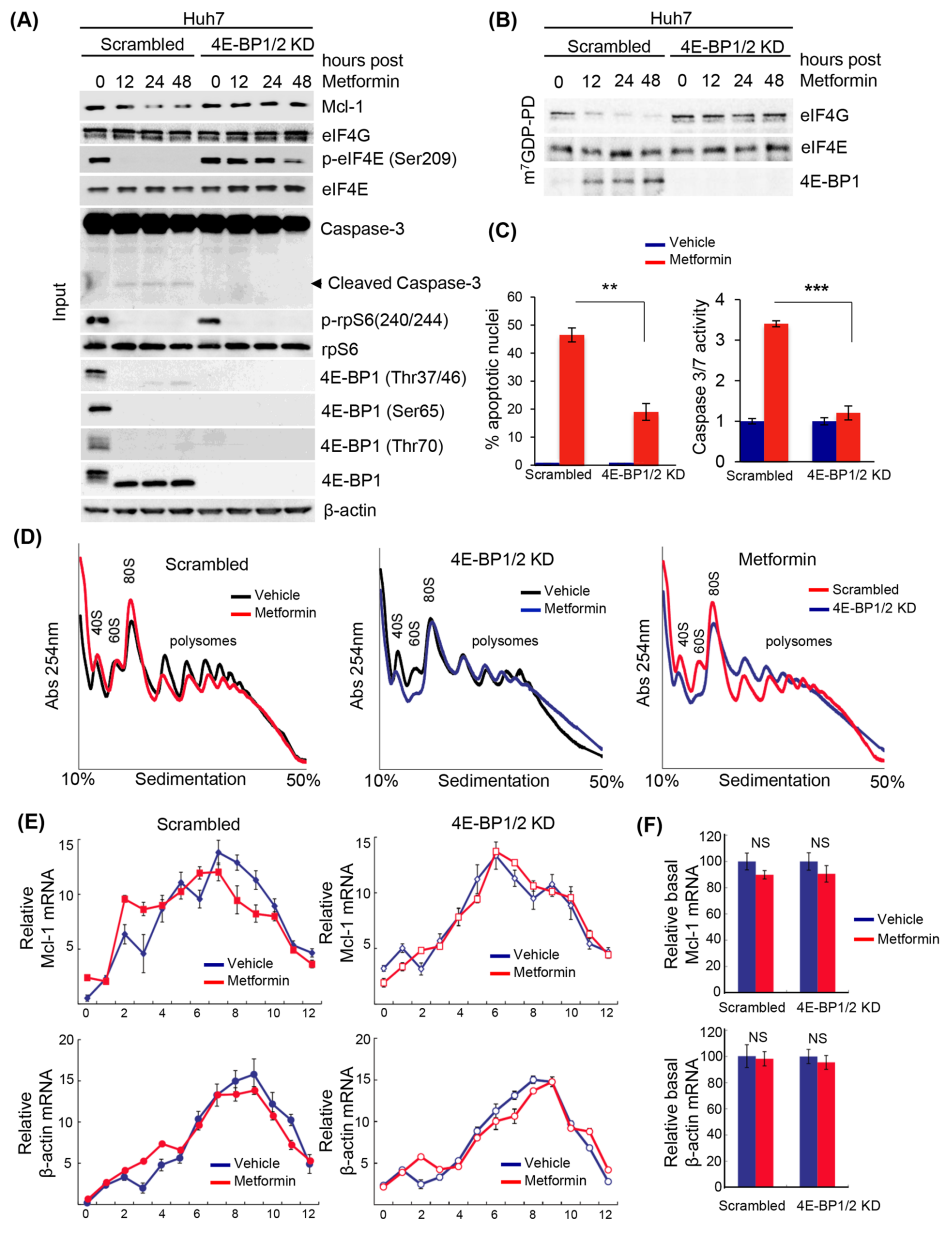
Metformin-induced apoptosis of liver cancer cells, and decreased translation of the anti-apoptotic mRNA *Mcl-1*, requires 4E-BP1/2 **A**. Western blot of indicated proteins in control (scrambled) and 4E-BP1/2 KD Huh7 cells treated with 10 mM metformin for 12, 24, or 48 hours. β-actin was used as a loading control; **B**. m^7^GDP pull-down assay. WT and 4E-BP1/2 KD Huh7 cells were treated with 10 mM metformin for the indicated time. Cells were lysed and cell lysates were incubated with m^7^GDP-agarose for 2 hours at 4°C. eIF4E, eIF4G and 4E-BP1 are shown by western blotting; **C**. Metformin induces apoptosis to a higher extent in scrambled versus 4E-BP1/2-depleted cells, as illustrated by a stronger increase in the percentage of apoptotic nuclei by DAPI assay (p=0.012), and caspase 3/7 positivity (p=0.0039) at 48 hours; **D**. Polysome profiles of scrambled (control) and 4E-BP1/2 KD Huh7 cells treated with vehicle or 10 mM metformin for 12 hours. Absorbance at 254 nm was continuously recorded; **E**. Distribution of *Mcl-1* and *β-actin* mRNAs fractionated across the density gradients from (D) was determined by RT-qPCR; **F**. Expression of total *Mcl-1* and *β-actin* mRNAs in cells treated as described in (D) was determined by RT-qPCR.

To assess the role of 4E-BPs on the apoptotic effect of metformin in HCC, we silenced 4E-BP1 and 4E-BP2 (4E-BP1/2) in Huh7 cells and examined the expression levels of Mcl-1 upon metformin treatment (Figure 4A). Metformin strongly decreased the phosphorylation of 4E-BP1 (on Thr37/46. Thr70 and Ser65), and reduced Mcl-1 protein levels in control cells. However, the expression of Mcl-1 in 4E-BP1/2 KD cells remained unchanged in the presence of metformin (Figure 4A). In addition, presence of cleaved caspase 3 (indicative of apoptosis), and reduction in phosphorylated eIF4E on Ser209 was observed only in control cells treated with metformin (Figure 4A). To determine whether metformin affects eIF4F complex formation in Huh7 cells, we performed a cap-column assay to measure the association of either 4E-BP1 or eIF4G with eIF4E. As shown in Figure 4B, metformin treatment resulted in decreased eIF4G and increased 4E-BP association with eIF4E. An increase in 4E-BP binding to eIF4E, and a corresponding decrease in eIF4G association is indicative of translational repression. Importantly, this effect did not occur in 4E-BP1/2 KD cells (Figure 4B). 4E-BP1/2 KD cells were more resistant to metformin-induced apoptosis compared to control cells, as illustrated by the presence of cleaved caspase 3 by western blotting (Figure 4A) as well as a reduced percentage of apoptotic nuclei and caspase 3/7 activity (Figure 4C). To address whether the reduction in Mcl-1 protein expression results from a translational repression of *Mcl-1* mRNA via 4E-BPs, we assessed the polysomal distribution of *Mcl-1* and *β-actin* mRNAs on control and 4E-BP1/2 KD cells treated with metformin or vehicle for 12 hours. Metformin treatment slightly decreased polysome formation in control cells, with 4E-BP1/2 KD cells being relatively unaffected by metformin (Figure 4D), as previously reported [72]. Notably, *Mcl-1* mRNA was partly shifted to lighter fractions after metformin treatment only in control cells (Figure 4E), with no difference in *β-actin* mRNA distribution between control and 4E-BP1/2 KD cells. Metformin did not affect total *Mcl-1* mRNA levels, confirming that the reduction in Mcl-1 protein expression is mediated at the level of mRNA translation rather than transcription at that time point (Figure 4F). Together with our polysome and mRNA distribution data, these results demonstrate that metformin-induced apoptosis and translational repression in Huh7 cells is mediated by 4E-BPs activation.

### Patient HCC tissue microarray reveals variability in the expression ratio between eIF4E and 4E-BP1

**Figure 5:**
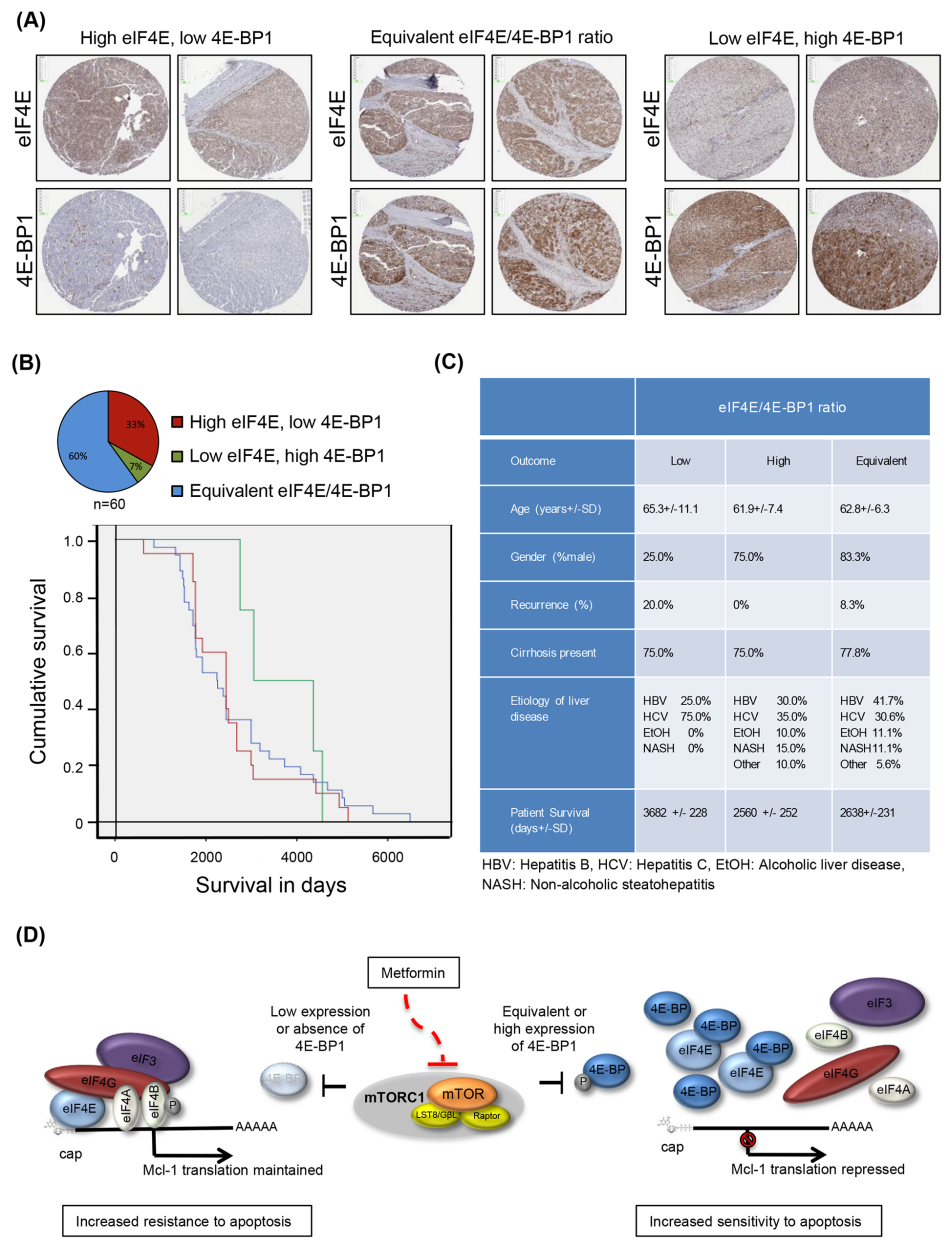
Patient HCC tumors demonstrate a variety of eIF4E and 4E-BP1 expression ratios **A**. and **B**. A patient microarray of 60 independent tumor tissues show a high eIF4E/4E-BP1 ratio in 33.0% of tumors, an equivalent eIF4E/4E-BP1 ratio in 60.0% of tumors, and a low eIF4E/4E-BP1 ratio in 7.0% of tumors. Kaplan-Meier curve demonstrates that tumors with a high eIF4E/4E-BP1 ratio are associated with decreased survival as compared to tumors with a low eIF4E/4E-BP1 ratio; **C**. Clinical and demographic characteristics of patients with Hepatocellular carcinoma tumors represented on tissue microarray, according to eIF4E/4E-BP1 ratio measured by immunohistochemistry; **D**. Proposed model for metformin induction of apoptosis via 4E-BPs. In cells with low to equivalent eIF4E/4E-BP1 ratio, metformin represses *Mcl-1* mRNA translation and induces apoptosis of liver cancer cells via activation of 4E-BPs, whereas in tumors with low or absence of 4E-BP1/2 expression (or potentially higher expression of eIF4E), *Mcl-1* mRNA translation is sustained and cells are resistant to metformin-induced apoptosis.

eIF4E is frequently over-expressed in malignant tissues including HCC [38, 39]. The expression ratio between eIF4E and its repressor 4E-BP1 has shown importance when treating with mTOR inhibitors, including indirect inhibitors such as metformin, as tumors with low eIF4E/4E-BP1 expression were reported to be more sensitive to mTOR inhibitors [40]; while tumors with high eIF4E/4E-BP1 showed increased resistance due to maintenance of mRNA translation of oncogenic and pro-survival factors. Thus determining whether levels of eIF4E versus 4E-BP1 vary in HCC tumors may be an important determinant prior to treatment. A patient HCC tissue microarray was used to probe the eIF4E/4E-BP1 ratio in HCC tumors by immunohistochemistry. Analysis of the patient HCC tissue microarray showed high, equivalent and low eIF4E/4E-BP1 expression across patient tumors (Figure 5A and 5B). Patient demographic and clinical characteristics, including age, gender, etiology of liver disease and presence of cirrhosis, were not different across the three categories (Figure 5C). Furthermore, tumor size and vascular invasion were also not different across the stratified samples. However, tumors with high eIF4E/4E-BP1 expression were more likely to be poorly differentiated (i.e. more aggressive), whereas tumors with a low eIF4E/4E-BP1 were more likely to be well differentiated (p=0.02). In addition, a lower overall survival of 2560 +/- 252 days after resection or transplant was found in patients with tumors having high eIF4E/4E-BP1 expression, while low eIF4E/4E-BP1 expression had an overall survival of 3682 +/- 228 days (Figure 5C). Patients with tumors having an equivalent eIF4E/4E-BP1 ratio had an overall survival of 2638+/-231 days after resection or transplant.

## DISCUSSION

In this study, we report that metformin induces apoptosis of liver cancer cells and inhibits translation of the mRNA encoding the anti-apoptotic protein Mcl-1. These effects require 4E-BPs, as silencing 4E-BP1/2 results in less repression of *Mcl-1* mRNA translation, and increased resistance to the apoptotic effect of metformin compared to control cells. These results have implications regarding the chemopreventive and potentially adjuvant role of metformin in HCC, especially given the recent expanding clinical literature. At the population level, large retrospective studies have found that metformin prevents development of HCC among diabetic patients [21–28] with chronic liver disease [29, 30]. A recent study has disproven the concern of lactic acidosis, revealing that cirrhotic patients may safely take metformin [41]. In fact, its use significantly extended survival in cirrhosis, with continuation of the medication decreasing risk of death by 57%. Hence, using metformin as a chemopreventive agent against incident HCC may be reasonable for patients with chronic liver disease.

Metformin has gained considerable interest as a preventive agent/adjunctive treatment through tumor “caloric restriction”, given its low cost and established safety profile [42, 43]. It is thought to affect tumor growth by two principal mechanisms: 1) through inhibition of mitochondrial oxidative phosphorylation, which activates AMPK thereby resulting in mTORC1 pathway inhibition, and 2) through decreased serum glucose, which inhibits IGF-R expression, thereby preventing downstream mTORC1 pathway activation in insulin-responsive cancers [44–50]. 4E-BP1 and eIF4E have been shown to be critical in the tumor generation driven by the Akt and N-Ras oncogenes in the genetic mouse model of HCC [36, 51]. Expression of 4E-BP1^4Ala^ (an unphosphorylatable form of 4E-BP1) significantly suppressed Akt/Ras-induced hepatocarcinogenesis through impaired mRNA translation [51]. In our study, metformin had a clear inhibitory and apoptotic effect on the Akt/Ras-induced tumors in the aforementioned genetic mouse model, with concomitant 4E-BP1 hypophosphorylation and decreased *Mcl-1* mRNA translation. By simple weight-based conversion, the dosing used in our mouse study would translate to 3000-4000 mg of metformin a day in a human (just above the maximum standard diabetic dosing), although pharmacokinetics will differ.

Recently, metformin has been shown to induce apoptosis of various cancer cell types, including lung, bladder and melanoma, as well as liver cancer [21, 52–65]. In oral cancer cells, metformin treatment was found to partly induce apoptosis through miR-26a-induced downregulation of Mcl-1. Recent studies [66, 67] have also reported that metformin, combined with aspirin or sodium dicholoroacetate respectively, suppresses Mcl-1 expression. Yue et al. suggested that this effect occurred partially through mTOR signaling and proteasome degradation. Metformin in combination with aspirin was found to synergistically induce apoptosis in a mouse model of pancreatic cancer by downregulating Mcl-1 [66], which strengthens the potential therapeutic application of our findings. Mcl-1 has the shortest half-life among all anti-apoptotic proteins, being highly unstable and needing active *Mcl-1* mRNA translation to maintain its expression [68]. When Mcl-1 expression is inhibited, induction of apoptosis has been shown in certain malignancies [69, 70]. mTORC1 inhibition in lymphomas dependent on the activation of this pathway resulted in decreased translation of *Mcl-1* mRNA and induction of apoptosis [35]. Our study establishes that induction of apoptosis by metformin in HCC, and repression of *Mcl-1* translation, requires the 4E-BPs.

The perturbation of the translatome by metformin has a certain degree of overlap with mTOR inhibitors [71]. A unique feature of metformin on the translatome is its preferential inhibition of a subset of mRNAs encoding proteins that promote proliferation and tumor growth [71]. Our study adds to this previous report by confirming that the apoptotic effect of metformin on liver cancer cells is mediated by the translational repressors 4E-BPs. This novel finding suggests a mechanism by which metformin induces apoptosis of liver cancer cells, and prevent establishment of tumors in patients with chronic liver disease. The eIF4E/4E-BP1 ratio is a key determinant of the response to mTOR inhibitors. Indeed, tumors with high eIF4E/4E-BP1 have decreased sensitivity to asTORi [40]. In our study, silencing 4E-BP1/2 (resulting in high eIF4E/4E-BP1) caused increased resistance to the apoptotic effect induced by metformin. Patient tumor samples in our study clearly exhibit a continuum of eIF4E/4E-BP1 ratios, and tumors with high eIF4E/4E-BP1 tend to be poorly differentiated and associated with lower overall survival. Conversely, the tumors exhibiting low eIF4E/4E-BP1 had the tendency to be well differentiated and correlated with better post-hepatectomy or transplant survival. It is expected that tumors with low eIF4E/4E-BP1 expression would be more sensitive to metformin, and those with high eIF4E/4E-BP1 would be more resistant due to maintenance of mRNA translation of oncogenic and pro-survival factors such as *Mcl-1* (Figure 5D). Hence, the various eIF4E/4E-BP1 ratios, reflecting tumor grade, have implications for the response to both metformin and asTORi.

In conclusion, metformin induces apoptosis of liver cancer cells and decreases the translation activity of the mRNA encoding for the anti-apoptotic protein Mcl-1. We found that these effects are mediated by 4E-BPs, as if these translational repressors are silenced, liver cancer cells are resistant to the apoptotic effect of metformin, and translation of *Mcl-1* mRNA is sustained. There are clearly certain limitations to our study, such as the inability to determine a clinically optimal inhibitory concentration for patients, given that *in vivo* pharmacokinetics vary from one species to another. In addition, a lengthier treatment of the genetic HCC mouse model might have resulted in a more dramatic effect as witnessed in a previous HCC mouse model subjected to 24 weeks of treatment [52]. Nonetheless, due to varying levels of 4E-BPs versus eIF4E in cancer tissues, our results propose an important determinant by which HCC tumors may be inherently resistant, or develop resistance, to metformin through downregulation of 4E-BPs.

## MATERIALS AND METHODS

### Ethics statement

Investigation has been conducted in accordance with the ethical standards and according to the Declaration of Helsinki and according to national and international guidelines and has been approved by the authors’ institutional review board.

### In vivo genetic HCC mouse model treated with metformin

A genetic mouse model of HCC was generated using hydrodynamic tail vein injection of transposons containing oncogenes myr-AKT1 and N-RasV12-containing plasmids concomitantly with the sleeping beauty transposase, as previously described [36]. These oncogenes belong to the mTOR and Ras/Raf/MAPK signaling axes shown to be activated in multiple tumor types. The plasmids were a kind gift from Dr. X. Chen at the University of California, San Francisco. FVB/N mice were obtained from Charles River (Wilmington, MA) and housed in the animal facility at the Mayo Clinic, Rochester, Minnesota. Two weeks following the hydrodynamic tail vein injections, male mice were randomized to 3 weeks of treatment with intraperitoneal injections of 0.2 ml of PBS only or metformin at 2 mg per mouse diluted in 0.2 ml of PBS. Mice were sacrificed at 5 weeks post-injection. After liver dissection, tumor nodules were isolated, fixed with formalin, and embedded in paraffin. The experimental protocol described in Figure 1A was approved by the Institutional Animal Care and Use Committee at the Mayo Clinic. Mice were fed a standard diet and monitored according to the animal committee's regulations. Slide sections (5 μm) were generated, and reviewed by a liver pathologist. These sections were immunostained for Mcl-1 (Abcam, 1:50), phospho-4E-BP1 (Cell signaling, 1:200), and cleaved caspase-3 antibody (Biocare medical, 1:50).

The immunohistochemical staining protocol was performed according to the manufacturer recommendations on an automated immunostainer (Discovery XT system, Ventana Medical Systems, Tucson, AZ). Antigen retrieval was performed with a proprietary citrated reagent. Primary antibodies as listed above were applied on the slides for 60 min at room temperature. Sections were then incubated with a secondary anti-rabbit biotinylated antibody. Streptavidin horseradish peroxidase, and 3, 3-diaminobenzidine were used according to the manufacturer's instructions (Ventana Medical Systems, Tucson, AZ). Finally, sections were counterstained with hematoxylin. The stained slides were analyzed using the Aperio ImageScope software (Vista, CA). A mouse-guided pen tool was used to outline the nodules on the slides. The pixel intensity reflected the amount of antibody bound, and is a measure of brightness, being proportional to the amount of light transmitted through the slide. The percentage of positive cells (% positivity) and total intensity of positive staining were quantified using the Positive Pixel Count Algorithm version 9.1. The intensity of positive staining was generated based on an intensity range of 0-255.

### Cell lines

The liver cancer cell lines Huh7 and HepG2 were obtained from the American Tissue Culture Collection (Manassas, VA), and maintained in Dulbecco's modified Eagle's medium with 25 mM glucose, supplemented with 10% fetal bovine serum and 100,000 IU/L penicillin as per the recommended protocols. The cell lines were passaged every 1-2 days, and kept in a 37°C in a 5% CO2, 95% air atmosphere incubator. Treatments included metformin (10 mM), sirolimus (20 nM), and active site mTOR inhibitors PP242 (1 μM), INK1341 (250 nM) and Torin1 (250 nM) for 72 hours.

### Cell viability assays

The Trypan blue exclusion assay was used to assess cell viability. Cells were seeded in 24-well plates (50,000 cells/well) over a 72-hour period with varying concentrations of metformin. Trypan blue staining excluded dead cells, and the number of viable cells was then determined by direct counting.

### Transduction of cell lines

Transduction of cell lines with lentiviruses containing shRNAs against 4E-BP1 and 4E-BP2 was performed in order to generate double knockdown (KD) cell lines. The control cell line was generated by transduction with lentivirus containing scrambled non-target shRNAs. Lentiviral vectors were obtained from Sigma (St. Louis, MO) [72]. shRNA vectors were co-transfected along with the lentivirus packaging plasmids PLP1, PLP2, and PLP-VSVG (Invitrogen) into Huh7 using Lipofectamine 2000 (Invitrogen). Transduced cells were selected the next day by incubating with puromycin in the medium for 48 hours (1 μg/ml, Sigma).

### Apoptosis assays

Both morphological and biochemical assays were performed to assess for apoptosis as previously described [73]. Huh7 and HepG2 cell lines were treated with PBS/DMSO (control), metformin or sirolimus. Treated and control cells were incubated with 2 μg/ml 4′,6-diamidine-2′-phenylindole dihydrochloride (DAPI) for 30 minutes at 37°C. The nuclei of apoptotic cells were stained with DAPI, with evidence of characteristic morphological changes in nuclear chromatin condensation and fragmentation were monitored by fluorescence microscopy (Nikon Eclipse TE200; Nikon, Tokyo, Japan). The same approach was employed to assess the degree of apoptosis in control and metformin-treated WT and 4E-BP1/2 KD Huh7 cells. The Caspase 3/7 fluorometric assay measured DEVD substrate cleavage and was performed according to the manufacturer's protocol using the Apo-One homogeneous caspase 3/7 assay (Promega, Madison, WI).

### Western blot analysis

Scrambled and 4E-BP1/2 KD Huh7 cells were treated with metformin 10 mM over a time course of 48 hours. Cells were lysed with RIPA lysis buffer with phosphatase inhibitors added at 0, 12, 24 and 48 hours post-treatment. Western blotting was carried out as previously described [74]. Antibodies against 4E-BP1 (Thr37/46, Ser65, Thr70), phospho-rpS6 (Ser240/244), Mcl-1, and phospho-eIF4E (Ser209) were purchased from Cell Signaling Technology (Danvers, MA). Antibody against eIF4E was purchased from Novus Biologicals (Littleton, CO). Bax (N-20) and Bad antibodies were from Santa Cruz Biotechnology (Dallas, TX). The antibody against β-actin was from Sigma-Aldrich (St. Louis, MO). Horseradish peroxidase-conjugated anti-rabbit IgG and anti-mouse IgG were from Santa Cruz Biotechnology.

### Cap-binding affinity assay

WT and 4E-BP1/2 KD Huh7 cells were treated with 10 mM metformin for the indicated time and lysed in lysis buffer [50 mM HEPES-KOH (pH 7.5), 150 mM KCl, 1 mM EDTA, 2mM DTT and 0.2 % Tween] containing protease inhibitors. Cell lysates were incubated with m^7^GDP-agarose for 2 hours at 4°C. Cap-binding proteins were washed four times using the lysis buffer, and then m^7^GDP-bound proteins were assessed by western blotting.

### Polysome analysis, RNA isolation and RT-qPCR

Scrambled and 4E-BP1/2 KD Huh7 cells were grown to 80% confluence. The cells were treated with vehicle (PBS) or 10 mM metformin for 12 hours. Polysome profile analysis was carried out as previously described [75].

The primers used for qPCR of *mcl-1* and *β-actin* were:

human *mcl1*_qPCR_5: GGACTGGCTAGTTAAA CAAAGAGG;

human *mcl1*_qPCR_3: CTTATTAGATATGCCA AACCAGCTC;

human *β-actin*_qPCR_5: AAGGCCAACCGTG AAAAGAT; and

human *β-actin*_qPCR_3: GTATGCCTCTGGT CGTACCAC.

### Tissue microarray construction and immunostaining

We established a list of patients having undergone liver transplant or partial hepatectomy for HCC between 1992 and 2009 using the McGill University Health Centre Liver cancer database. Only those patients who were eligible for liver transplant (i.e. for HCC within Milan criteria: a single tumor less than 5 cm in diameter, or less than/equal to 3 tumors less than 3 cm in diameter) or partial hepatectomy for HCC in patients with well compensated liver function (HCC that had arisen in the context of chronic viral hepatitis or Child-Pugh A cirrhosis) were included in the study. Thus, none of the patients had HCC beyond Stage 2. Exclusion criteria were the use of transarterial chemoembolization, radiofrequency ablation, any other locoregional therapies or chemotherapy prior to banking of pathology specimens, as the resulting necrotic samples would not appropriately reflect protein expression. Patients were approached and consented for use of their HCC tissue samples, with the ethics protocol having been approved by the McGill University Health Centre Institutional Review Board.

Slides of liver resections were reviewed by a liver pathologist, and HCC nodules were identified. The corresponding tissue blocks were marked for use in the Tissue Microarray (TMA), and sample cores were removed from the tissue blocks. These cores were incorporated into a TMA using a microarray instrument. The tissue microarray slides were subjected to immunohistochemical staining for eIF4E and 4E-BP1, as per the protocol described above. Grading of immunohistochemical staining was performed independently by 3 investigators (MB, TA, DZ) as follows: no/little staining, moderate staining, and strong staining. The eIF4E to 4E-BP1 ratio for each nodule was determined by relative staining intensity, leading to 3 ratio categories: (1) high eIF4E/4E-BP1 ratio, (2) equivalent eIF4E/4E-BP1 ratio, and (3) low eIF4E/4E-BP1 ratio. These ratios were assessed for correlations with clinical and demographic characteristics, tumor pathological features (grade, size), as well as overall survival following resection/transplant.

### Statistical analysis

Statistical analysis to calculate p-values was performed using unpaired two-tail student t-test. Standard Errors (SEs) for data are represented using error bars on all graphs.
